# Novel isoguanine derivative of unlocked nucleic acid—Investigations of thermodynamics and biological potential of modified thrombin binding aptamer

**DOI:** 10.1371/journal.pone.0197835

**Published:** 2018-05-24

**Authors:** Weronika Kotkowiak, Tomasz Czapik, Anna Pasternak

**Affiliations:** 1 Department of Nucleic Acids Bioengineering, Institute of Bioorganic Chemistry, Polish Academy of Sciences, Noskowskiego, Poznan, Poland; 2 Department of Structural Chemistry and Biology of Nucleic Acids, Institute of Bioorganic Chemistry, Polish Academy of Sciences, Noskowskiego, Poznan, Poland; Consiglio Nazionale delle Ricerche, ITALY

## Abstract

Thrombin binding aptamer (TBA), is a short DNA 15-mer that forms G-quadruplex structure and possesses anticoagulant properties. Some chemical modifications, including unlocked nucleic acids (UNA), 2′-deoxy-isoguanosine and 2′-deoxy-4-thiouridine were previously found to enhance the biological activity of TBA. In this paper, we present thermodynamic and biological characteristics of TBA variants that have been modified with novel isoguanine derivative of UNA as well as isoguanosine. Additionally, UNA-4-thiouracil and 4-thiouridine were also introduced simultaneously with isoguanine derivatives. Thermodynamic analysis indicates that the presence of isoguanosine in UNA or RNA series significantly decreases the stability of G-quadruplex structure. The highest destabilization is observed for substitution at one of the G-tetrad position. Addition of 4-thiouridine in UNA or RNA series usually decreases the unfavorable energetic cost of the presence of UNA or RNA isoguanine. Circular dichroism and thermal denaturation spectra in connection with thrombin time assay indicate that the introduction of UNA-isoguanine or isoguanosine into TBA negatively affects G-quadruplex folding and TBA anticoagulant properties. These findings demonstrate that the highly-ordered structure of TBA is essential for inhibition of thrombin activity.

## Introduction

Anticoagulants belong to a class of compounds that influence blood coagulation by prolonging clotting time. From the medical point of view, anticoagulants prevent the formation of blood clots or inhibit the growth of formed clots, thus decreasing the risk of emboli formation in vessels of organs [1,2]. Anticoagulants are commonly used as drugs for the prevention and/or treatment of heart attack, stroke, deep vein or stent thrombosis, pulmonary embolism, cancer (as supportive therapy) and other pathologies [3,4]. Unfortunately, currently used anticoagulants show many side effects which include prolonged bleeding or even fatal bleeding.

**Fig 1:**
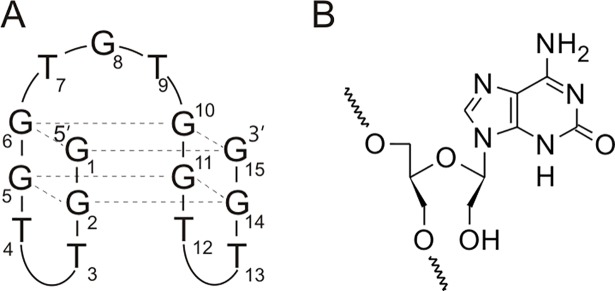
Structure of thrombin binding aptamer (A) and novel isoguanine derivative of UNA (B).

Anticoagulants may reduce the activity of clotting factors directly or indirectly. One of the direct thrombin inhibitors is thrombin binding aptamer (TBA), a DNA 15-mer [5,6]. This oligonucleotide forms an intramolecular, antiparallel G-quadruplex structure with a chair-like conformation containing two G-tetrads connected by three edge-wise loops (Fig 1A). According to NMR and X-ray studies, TBA interacts with the positively charged exosite I of thrombin *via* two shorter TT loops, thus inhibiting enzyme activity [6,7]. Previous reports indicate that there are a few chemical modifications, among those unlocked nucleic acids (UNA), 4-thio-2′-deoxyuridine and 2′-deoxy-isoguanosine, that are able to increase thermodynamic and/or anticoagulant properties of the aptamer [8–10].

Isoguanosine (iG) is an unnatural nucleoside that pairs with isocytidine (iC) *via* three hydrogen bonds with the strength comparable to regular G-C interactions [11]. Interestingly, hybridization of isoguanosine with cytidine (iG-C) was found to be isoenergetically similar to A-U base pair. The strong interactions between iG and iC can be potentially applied in the quantitative hybridization assay to prevent non-specific signal amplification, in PCR techniques of DNA containing unnatural base pairs, in nanotechnology to construct more intricate structures or in antisense therapy where iG-iC base pair might increase the specificity of aptamers and decoys [12,13].

4-Thiouridine (s4U) is a photoreactive uridine analogue that was found to naturally occur in bacterial and archaeal tRNAs and play a role in protecting cells from near-UV light [14]. Moreover, in eukaryotes this thiolated nucleoside is successfully applied as metabolic label, upon its random incorporation during transcription [15]. Previously reported thermodynamic studies indicate that s4U slightly increases thermodynamic stability of A-U base pair [16]. Notably, the presence of s4U significantly stabilizes G-U wobble base pair, thus decreasing base pairing specificity of A-U vs. G-U. Due to natural occurrence of 4-thiouridine and its crosslinking properties, s4U was used as molecular tool in many analytical strategies [15].

It was found that certain UNA residues at position T^7^ of TBA increase thermodynamic stability of G-quadruplex and improve anticoagulant potential of the aptamer [8]. Similarly, substitution of 4-thio-2′-deoxyuridine at positions T^3^, T^7^, T^9^ and T^13^ in TBA was found to inhibit thrombin-catalyzed fibrin clotting 2-fold compared to unmodified TBA [9]. Moreover, based on colorimetric assay, Nallagatla *et al*. performed screening of the oligonucleotide library containing all possible substitutions of 2′-deoxy-isoguanosine and indicated that its presence at positions G^1^, G^8^ or G^10^ in TBA improves the aptamer-thrombin interaction [10].

Stimulated by the previously published results, we decided to identify potential of novel chemical modifications that alter both sugar and heterocyclic base moieties known to be favorable for TBA (5′GGTTGGTGTGGTTGG3ʹ) biological activity. Recently, we reported anticoagulant and antiproliferative properties of modified TBA variants containing a 4-thiouracil derivative of UNA (UNA-s4U) [17]. Herein, we present chemical synthesis of UNA-isoguanine (iG^U^) a novel UNA derivative (Fig 1B), its incorporation into TBA and a comprehensive thermodynamic and biological characteristics of novel TBA variants. Moreover, we also analyzed TBA variants with isoguanosine as well as with mixed 4-thiouridine and isoguanosine substitutions in RNA (s4U^R^ or iG^R^) or UNA (s4U^U^ or iG^U^) series.

## Materials and methods

### Chemical synthesis of UNA-isoguanine phosphoramidite

**Fig 2:**
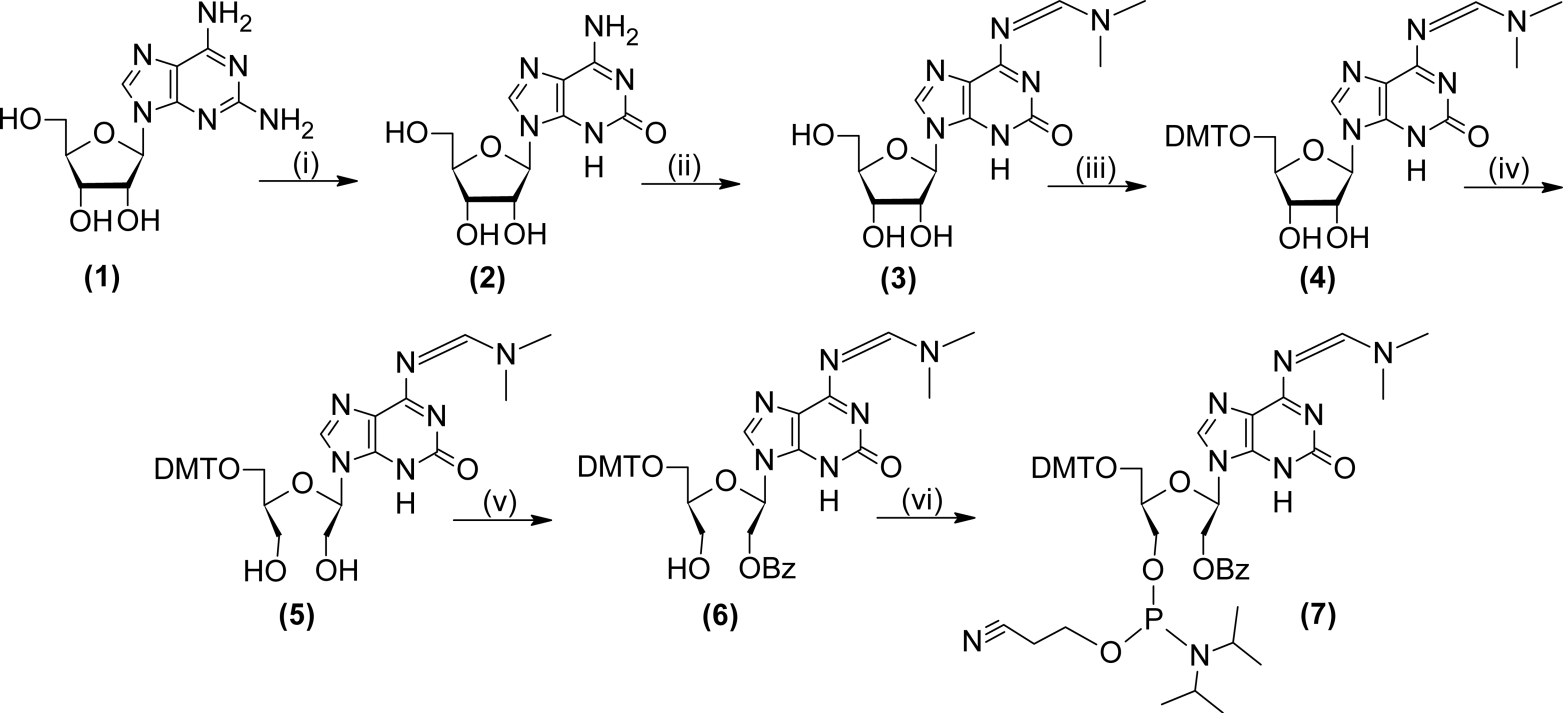
Chemical synthesis of UNA-isoguanine phosphoramidite. Reagents: (i) LiNO_2_, AcOH, H_2_O; (ii) *N*,*N*-dimethylformamide dimethyl acetal, DMF; (iii) DMTCl, Py; (iv) NaIO_4_, 1,4-dioxane/water; NaBH_4_, 1,4-dioxane/water; (v) BzCl, DCM, -70°C; (vi) 2-cyanoethyl-*N*,*N*,*N*′,*N*′-tetraisopropylphosphordiamidite, tetrazole, MeCN.

The synthesis of protected UNA-isoguanine phosphoramidite (Fig 2) was performed according to general procedure of the synthesis of UNA phosphoramidites with some modifications [18]. 2,6-Diaminopurine riboside (**1**) was treated with lithium nitrite and acetic acid to yield isoguanosine (**2**). Treatment of compound (**2**) with *N*,*N*-dimethylformamide dimethyl acetal resulted in the *N*6 protected derivative (**3**) which was converted with dimethoxytrityl chloride into 5′-O-4,4′-dimethoxytrityl derivative (**4**). Compound (**4**) was treated with sodium periodate followed by sodium borohydride to produce UNA derivative (**5**). Selective benzoylation of (**5**) at O2′ was achieved by addition of benzoyl chloride at decreased temperature accordingly to the previously published protocol [18]. Treatment of the resulting compound (**6**) with 2-cyanoethyl-*N*,*N*,*N*′,*N*′-tetraisopropylphosphordiamidite gave the final UNA-isoguanine in overall yield *ca*. 17%. Protocol for the synthesis of UNA-isoguanine phosphoramidite is described in S1 File. The ^1^H and ^13^C NMR spectra were recorded on a 500 MHz (11.74 T) AVANCE III Bruker spectrometer. Thin layer chromatography was conducted on Merck silica gel 60 F254 glass plates. Silica gel column chromatography was performed using Merck TLC gel H 60.

### Chemical synthesis of oligonucleotides

All oligonucleotides were synthesized on an automated RNA/DNA synthesizer using standard phosphoramidite chemistry [19]. The deprotection and purification of unmodified oligoribonucleotides or TBA variants containing UNA-iG and RNA-iG were performed according to general procedures. Deprotection of TBA variants modified simultaneously with RNA-s4U and RNA-iG or UNA-s4U and UNA-iG was performed by the treatment with methanol/aqueous ammonia solution (1:1 v/v) at room temperature for 48 h. The purification was accomplished according to previously published procedures [20]. The composition of all oligonucleotides was confirmed by MALDI-TOF mass spectrometry.

### UV melting analysis

Oligonucleotides were dissolved in a buffer containing 100 mM potassium chloride, 20 mM sodium cacodylate and 0.5 mM Na_2_EDTA, pH 7.0. Single-stranded oligonucleotide concentrations were calculated based on the absorbance measured above 80°C [21] and the extinction coefficients were calculated, using Oligo Calculator (ribotask.com). The samples were denatured at 95°C for 5 min and then cooled to room temperature overnight. The measurements were performed for nine different concentrations of oligonucleotide in the concentration range of 10−4–10^-6^ M. Absorbance versus temperature curves were obtained using the UV melting method at 295 nm in the temperature range of 4–90°C with a heating rate of 0.2°C/min on a JASCO V-650 spectrophotometer equipped with a thermoprogrammer. Melting curves were analyzed using the MeltWin 3.5 software by van’t Hoff analysis [22]. The thermodynamic parameters were determined by nonlinear curve fitting, averaging ΔH° and ΔS° from fits of individual melting curves at different concentrations. For TBA variants presented in S1 Table the alternative method was additionally applied *i*.*e*. plots of 1/T_m_ vs lnC_T_ were fitted to the following equation:

$$T_{m}^{-1}=(R/\Delta H{}^{\circ})lnC_{T}+\Delta S{}^{\circ}/\Delta H{}^{\circ}$$

where C_T_ is the total oligonucleotide strand concentration and *R* is the gas constant. Both methods assume a two-state model. Agreement within 15% of ΔH° obtained from both methods was indicative of the two-state approximation.

Melting temperatures calculated for a 10^−4^ M concentration of oligonucleotide are denoted by T_M_, and melting points for any other concentration of oligonucleotide are denoted by T_m_.

### Circular dichroism spectra

CD spectra were recorded on a JASCO J-815 spectropolarimeter. The oligonucleotides were dissolved in a buffer containing 100 mM potassium chloride, 20 mM sodium cacodylate and 0.5 mM Na_2_EDTA, pH 7.0, to achieve a sample concentration of 3.0 μM. All samples were denatured at 95°C for 5 min and then slowly cooled to room temperature overnight prior to data collection. The spectra were recorded in triplicate at 37°C in the 205–320 nm wavelength range. The buffer spectrum was subtracted from the sample spectra. The data analysis was performed using the Origin 8.0 software.

### Thermal difference spectra

The TDS measurements were performed on a JASCO V-650 spectrophotometer equipped with a thermoprogrammer. The oligonucleotides were dissolved in a buffer containing 100 mM potassium chloride, 20 mM sodium cacodylate and 0.5 mM Na_2_EDTA, pH 7.0, to achieve a sample concentration of 0.1 μM. Absorbance spectra were recorded in triplicate at 4°C and 90°C in the 220–335 nm wavelength range. The scan speed was 1000 nm/min with a 1 nm data interval. Thermal difference spectra were obtained by subtraction of the low temperature absorbance spectra from the high temperature absorbance spectra using the Origin 8.0 software. The differential spectra were normalized by dividing the data by its maximum value [23].

### Thrombin time assay

The thrombin time assay was performed using commercially available Dia-TT kit (DIAGON®) and coagulometer K-3002 Optic. Each reaction mixture consisted of TBA variant (0.33 μM) dissolved in 100 μl Dia-TT reagent. The reaction mixture was incubated at 37°C for 5 min and put into sample well of coagulometer K-3002 Optic. Next, the 100 μl of citrate plasma was added. Each TBA variant was analyzed using plasma samples derived from five healthy volunteers. The anticoagulant effect was obtained by subtraction of time needed for plasma clotting in the presence of the aptamers from time needed for clotting of poor plasma.’

## Results and discussion

### Thermodynamic analysis of TBA variants containing UNA-iG or RNA-iG

TBA variants containing single and multiple substitution of modified residues were synthesized and analyzed to determine the influence of RNA-iG or UNA-iG on the G-quadruplex thermodynamic stability, folding topology and biological properties. The positions of modification G^1^, G^8^ and G^10^ were selected according to a previous report indicating these positions as the most favorable for anticoagulant properties of TBA modified with 2′-deoxy-isoguanosine [10].

Thermodynamic analysis showed that the presence of UNA-iG or RNA-iG at positions G^1^ and G^10^, involved in G-quartet formation, significantly destabilize G-quadruplex structure (Table 1). Modification of G-tetrad at terminal position (G^1^) by UNA-iG or RNA-iG decreases G-quadruplex thermodynamic stability in a comparable manner (ΔΔG°_37_ = 3.2–3.3 kcal/mol). In contrast, the presence of UNA-iG within internal position G^10^ of the G-quartet destabilizes G-quadruplex more than RNA-iG (ΔΔG°_37_ = 3.39 kcal/mol for UNA-iG and 2.13 kcal/mol for RNA-iG). Despite previous reports suggesting that mixed G-quartets containing both isoguanosine and guanosine can be formed [24] in this case, large destabilization of the G-quadruplex structure suggests that the unfavorable effect might be a consequence of a change in the spatial arrangement of the amino and carbonyl functional groups within RNA-iG and UNA-iG as well as the *anti*-conformation of N-glyosidic bond relative to *syn*-oriented 2′-deoxyguanosines at G^1^ and G^10^. Nevertheless, in the future further structural studies are needed to confirm the above theory.

**Table 1:** Thermodynamic stability of TBA variants modified with RNA-iG (iG^R^) or UNA-iG (iG^U^)^a^.

Position of modification	Sequence (5ʹ-3ʹ)	Average of curve fits					
		ΔG˚_37_ (kcal/mol)	T_M_ (˚C)	ΔΔG˚_37_ (kcal/mol)	ΔT_M_ (˚C)	ΔΔG˚_37_ (kcal/mol)	ΔT_M_ (˚C)
	GGTTGGTGTGGTTGG	-1.74±0.02	50.7	0	0		
G^1^	**iG^R^**GTTGGTGTGGTTGG	1.49±0.48	19.9	3.23	-30.8	0	0
G^1^	**iG^U^**GTTGGTGTGGTTGG	1.55±0.07	20.7	3.29	-30.0	0.06	0.8
G^8^	GGTTGGT**iG^R^**TGGTTGG	-0.51±0.02	41.6	1.23	-9.1	0	0
G^8^	GGTTGGT**iG^U^**TGGTTGG	0.21±0.01	35.1	1.95	-15.6	0.72	-6.5
G^10^	GGTTGGTGT**iG^R^**GTTGG	0.39±0.05	31.9	2.13	-18.8	0	0
G^10^	GGTTGGTGT**iG^U^**GTTGG	1.65±0.33	23.8	3.39	-26.9	1.26	-8.1
G^1^, G^8^	**iG^R^**GTTGGT**iG^R^**TGGTTGG	1.36±0.14	22.3	3.10	-28.4		
G^1^, G^8^	**iG^U^**GTTGGT**iG^U^**TGGTTGG	n.d.	<15.0	-	-	-	-
G^8^, G^10^	GGTTGGT**iG^R^**T**iG^R^**GTTGG	0.31±0.01	34.1	2.05	-16.6		
G^8^, G^10^	GGTTGGT**iG^U^**T**iG^U^**GTTGG	n.d.	<15.0	-	-	-	-
G^1^, G^10^	**iG^R^**GTTGGTGT**iG^R^**GTTGG	n.d.	<15.0	-	-	-	-
G^1^, G^10^	**iG^U^**GTTGGTGT**iG^U^**GTTGG	n.d.	<15.0	-	-	-	-
G^1^, G^8^, G^10^	**iG^R^**GTTGGT**iG^R^**T**iG^R^**GTTGG	n.d.	<15.0	-	-	-	-
G^1^, G^8^, G^10^	**iG^U^**GTTGGT**iG^U^**T**iG^U^**GTTGG	n.d.	<15.0	-	-	-	-

^a^ buffer: 100 mM KCl, 20 mM sodium cacodylate, 0.5 mM EDTA(Na)2, pH 7.0

n.d.–not determined, detailed thermodynamic analysis is presented in S3 Table

The substitution of TBA loop at position G^8^ by UNA-iG or RNA-iG also destabilizes G-quadruplex structure, but the effect is reduced in comparison to positions G^1^ and G^10^ that are involved in G-quartet formation. The presence of UNA-iG at G^8^ decreases G-quadruplex thermodynamic stability by 1.95 kcal/mol, whereas RNA-iG causes destabilization mounting to 1.23 kcal/mol. According to some structural analysis of unmodified TBA, the carbonyl group of 2′-deoxyguanosine at G^8^ may form a hydrogen bond with the 2′-deoxyguanosine at G-quartet [25–28]. Therefore, the unfavorable effect induced by the presence of UNA-iG or RNA-iG at G^8^ might result from H-bond disruption between the nucleobase at G^8^ and the G-quadruplex core. This is most likely due to opposite carbonyl and amino group arrangement in isoguanosine relative to guanosine. Notably, UNA-iG at position G^8^ decreases TBA thermodynamic stability more than RNA-iG at the same position. The increased flexibility of UNA-iG at G^8^ compared to RNA-iG might additionally hinder a more favorable nucleobase orientation contributing to a higher destabilization for the UNA than RNA modification. A similar trend was observed for G^10^ but not for the G^1^ position. This is probably due to the internal (G^8^ and G^10^) vs. terminal (G^1^) location, which is less and more tolerant towards structural changes, respectively [29,30].

As expected, the simultaneous substitution of UNA-iG or RNA-iG at more than one of the TBA G^1^, G^8^ and G^10^ positions caused significant destabilization that prevented thermodynamic analysis of most multi-modified G-quadruplex variants (Table 1).

Notably, the Gibbs free energy of almost all analyzed TBA variants possesses positive values, with the exception of the oligonucleotide substituted by RNA-iG at G^8^. This indicates that the unfolded G-quadruplex form is the predominant species at physiological temperature for the majority of modified oligonucleotides.

### Thermodynamic analysis of TBA variants containing UNA-iG or RNA-iG and UNA-s4U or RNA-s4U

In the light of reports that indicate 2′-deoxy-isoguanosine, 2′-deoxy-4-thiouridine and UNA as the residues having potential to improve the anticoagulant properties of TBA [8–10], we decided to analyze TBA variants that contain simultaneously UNA-iG and UNA-s4U as well as RNA-iG and RNA-s4U. UNA-s4U or RNA-s4U were substituted at positions T^3^, T^7^, T^9^, T^12^ or T^13^ and UNA-iG or RNA-iG at G^1^, G^8^ and G^10^ in TBA. These positions were selected based on earlier publications, which indicated the positions as most biologically favorable [9,10].

The presence of RNA-s4U at positions T^9^ or T^12^ with RNA-iG at G^1^ mitigated an unfavorable thermodynamic effect triggered by isoguanosine having a ΔΔG°_37_ = 1.83 kcal/mol for G^1^, T^9^ and 1.85 kcal/mol for G^1^, T^12^ (Table 2). On the contrary, the presence of UNA-s4U at T^3^ or T^7^ and UNA-iG at G^1^ as well as RNA-s4U at T^7^ and RNA-iG at G^1^ does not significantly reduce destabilization caused by isoguanine derivatives. Moreover, for TBA variants containing UNA-iG or RNA-iG at G^1^ together with UNA-s4U at T^9^, T^12^ or RNA-s4U at T^7^ the destabilization of G-quadruplex structure was even higher relative to the TBA modified with a single isoguanosine in UNA or RNA series at G^1^ (ΔΔG°_37_ = 3.8–4.0 kcal/mol). Unfortunately, we were unable to analyze the thermodynamics of TBA variants with substitutions at positions T^13^ and G^1^ by UNA-s4U and UNA-iG or RNA-s4U and RNA-iG due to extremely large destabilization.

**Table 2:** Thermodynamic stability of TBA variants modified with RNA-iG and RNA-s4U (iG^R^,s4U^R^) or UNA-iG and UNA-s4U (iG^U^,s4U^U^)^a^.

Position of modification	Sequence (5ʹ-3ʹ)	Average of curve fits					
		ΔG˚_37_ (kcal/mol)	T_M_ (˚C)	ΔΔG˚_37_ (kcal/mol)	ΔT_M_ (˚C)	ΔΔG˚_37_ (kcal/mol)	ΔT_M_ (˚C)
	GGTTGGTGTGGTTGG	-1.74±0.02	50.7	0	0		
G^1^, T^3^	**iG^R^**G**s4U^R^**TGGTGTGGTTGG	1.39±0.06	29.0	3.13	-21.7	0	0
G^1^, T^3^	**iG^U^**G**s4U^U^**TGGTGTGGTTGG	1.48±0.07	20.6	3.22	-30.1	0.09	-8.4
G^1^, T^7^	**iG^R^**GTTGG**s4U^R^**GTGGTTGG	2.18±0.22	17.1	3.92	-33.6	0	0
G^1^, T^7^	**iG^U^**GTTGG**s4U^U^**GTGGTTGG	1.33±0.06	22.7	3.07	-28	-0.85	5.6
G^1^, T^9^	**iG^R^**GTTGGTG**s4U^R^**GGTTGG	0.09±0.01	36.0	1.83	-14.7	0	0
G^1^, T^9^	**iG^U^**GTTGGTG**s4U^U^**GGTTGG	2.21±0.19	20.0	3.95	-30.7	2.12	-16.0
G^1^, T^12^	**iG^R^**GTTGGTGTGG**s4U^R^**TGG	0.11±0.02	35.9	1.85	-14.8	0	0
G^1^, T^12^	**iG^U^**GTTGGTGTGG**s4U^U^**TGG	2.04±0.11	19.4	3.78	-31.3	1.93	-16.5
G^1^, T^13^	**iG^R^**GTTGGTGTGGT**s4U^R^**GG	n.d.	<15.0	-	-	-	-
G^1^, T^13^	**iG^U^**GTTGGTGTGGT**s4U^U^**GG	n.d.	<15.0	-	-	-	-
T^3^, G^8^	GG**s4U^R^**TGGT**iG^R^**TGGTTGG	-0.81±0.03	44.1	0.93	-6.6	0	0
T^3^, G^8^	GG**s4U^U^**TGGT**iG^U^**TGGTTGG	0.19±0.02	35.2	1.93	-15.5	1	-8.9
T^7^, G^8^	GGTTGG**s4U^R^iG^R^**TGGTTGG	-0.88±0.04	44.4	0.86	-6.3	0	0
T^7^, G^8^	GGTTGG**s4U^U^iG^U^**TGGTTGG	0.10±0.07	36.1	1.84	-14,6	0.98	-8.3
G^8^, T^9^	GGTTGGT**iG^R^s4U^R^**GGTTGG	-0.81±0.03	44.5	0.93	-6.2	0	0
G^8^, T^9^	GGTTGGT**iG^U^s4U^U^**GGTTGG	0.71±0.03	30.4	2.45	-20.3	1.52	-14.1
G^8^, T^12^	GGTTGGT**iG^R^**TGG**s4U^R^**TGG	-0.85±0.04	44.1	0.89	-6.6	0	0
G^8^, T^12^	GGTTGGT**iG^U^**TGG**s4U^U^**TGG	0.17±0.02	35.4	1.91	-15.3	1.02	-8.7
G^8^, T^13^	GGTTGGT**iG^R^**TGGT**s4U^R^**GG	0.36±0.10	33.6	2.10	-17.1	0	0
G^8^, T^13^	GGTTGGT**iG^U^**TGGT**s4U^U^**GG	0.95±0.01	28.0	2.69	-22.7	0.59	-5.6
T^3^, G^10^	GG**s4U^R^**TGGTGT**iG^R^**GTTGG	-0.19±0.04	38.5	1.55	-12.2	0	0
T^3^, G^10^	GG**s4U^U^**TGGTGT**iG^U^**GTTGG	1.87±0.17	22.3	3.61	-28.4	2.06	-16.2
T^7^, G^10^	GGTTGG**s4U^R^**GT**iG^R^**GTTGG	-0.21±0.02	38.8	1.53	-11.9		
T^9^, G^10^	GGTTGGTG**s4U^R^iG^R^**GTTGG	-0.54±0.02	42.6	1.2	-8.1		
G^10^, T^12^	GGTTGGTGT**iG^R^**G**s4U^R^**TGG	-0.20±0.02	38.7	1.54	-12.0	0	0
G^10^, T^12^	GGTTGGTGT**iG^U^**G**s4U^U^**TGG	1.61±0.09	21.3	3.35	-29.4	1.81	-17.4
G^10^, T^13^	GGTTGGTGT**iG^R^**GT**s4U^R^**GG	0.60±0.02	30.6	2.34	-20.1		
G^1^, T^3^, T^7^, T^9^, T^13^	**iG^R^**G**s4U^R^**TGG**s4U^R^**G**s4U^R^**GGT**s4U^R^**GG	0.99±0.08	24.7	2.73	-26.0		
T^3^, T^7^, G^8^, T^9^, T^13^	GG**s4U^R^**TGG**s4U^R^iG^R^s4U^R^**GGT**s4U^R^**GG	-0.50±0.09	42.0	1.24	-8.7		
T^3^, T^7^, T^9^, G^10^, T^13^	GG**s4U^R^**TGG**s4U^R^**G**s4U^R^iG^R^**GT**s4U^R^**GG	-0.17±0.04	38.7	1.57	-12.0		
G^1^, T^3^, T^7^, G^8^, T^9^, G^10^, T^13^	**iG^R^**G**s4U^R^**TGG**s4U^R^iG^R^s4U^R^iG^R^**GT**s4U^R^**GG	n.d.	<15	-	-	-	-
G^1^, T^3^, T^7^, G^8^, T^9^, G^10^, T^13^	**iG^U^**G**s4U^U^**TGG**s4U^U^iG^U^s4U^U^iG^U^**GT**s4U^U^**GG	n.d.	<15	-	-	-	-

^a^ buffer: 100mM KCl, 20mM sodium cacodylate, 0.5 mM EDTA(Na)2, pH 7.0

n.d.–not determined, detailed thermodynamic analysis is presented in S4 Table

Simultaneous substitutions of TBA at positions G^8^ by UNA-iG or RNA-iG and T^3^, T^7^, T^9^ or T^12^ by UNA-s4U or RNA-s4U usually caused an increase in G-quadruplex thermodynamic stability compared to TBA modified with single UNA-iG or RNA-iG at G^8^ (Table 2). TBA variants with RNA-iG at G^8^ and RNA-s4U at T^3^, T^7^, T^9^ or T^12^ were found to be the most stable. In contrast, the most unfavorable effects were observed for TBA variants containing UNA-iG at G^8^ and UNA-s4U at T^9^ or T^13^ as well as RNA-iG at G^8^ and RNA-s4U at T^13^.

Similarly, substitution of positions T^3^, T^7^, T^9^ or T^12^ by UNA-s4U or RNA-s4U reduced some unfavorable thermodynamic effects caused by UNA-iG or RNA-iG at position G^10^. However, the presence of UNA-s4U at T^3^ and T^12^ together with UNA-iG at G^10^ causes significant destabilization (ΔΔG°_37_ = 3.61 kcal/mol for T^3^ and G^10^ and 3.35 kcal/mol for T^12^ and G^10^). Interestingly, introduction of UNA-s4U at position T^7^, T^9^ or T^13^ and UNA-iG at G^10^ changed intramolecular to intermolecular folding of TBA (S1 Table). Thus, the direct comparison of Gibbs free energy of those variants was not possible. Nevertheless, the analysis of T_M_ values indicates these variants do not form G-quadruplex structure at physiological temperature. A similar trend of molecularity change was observed with UNA-iG residue at G^1^, G^8^ or G^10^ and four UNA-s4U residues at T^3^, T^7^, T^9^ and T^13^.

In general, the thermodynamic effects observed for multiple modifications of TBA are the sum of single modification effects of UNA/RNA-iG and UNA/RNA-s4U. These findings stay in great accordance with the influence of UNA-s4U and RNA-s4U on TBA thermodynamic stability recently reported by our group [17].

### CD spectroscopy

Circular dichroism is a commonly used technique to investigate structural geometry of various nucleic acid structures. Particularly, when studying G-quadruplexes, it allows differentiation between antiparallel and parallel folding topology, thus one can identify any alteration in this molecular process. The specific CD pattern might be a result of the RNA or DNA strands orientation, but also the stacking arrangement of guanines within G-tetrads [31]. Usually, parallel G-quadruplexes are characterized by CD spectra containing positive band near 265 nm and a negative peak around 240 nm, whereas antiparallel G-quadruplexes exhibit positive signal around 295 nm and a negative band near 265 nm [32].

**Fig 3:**
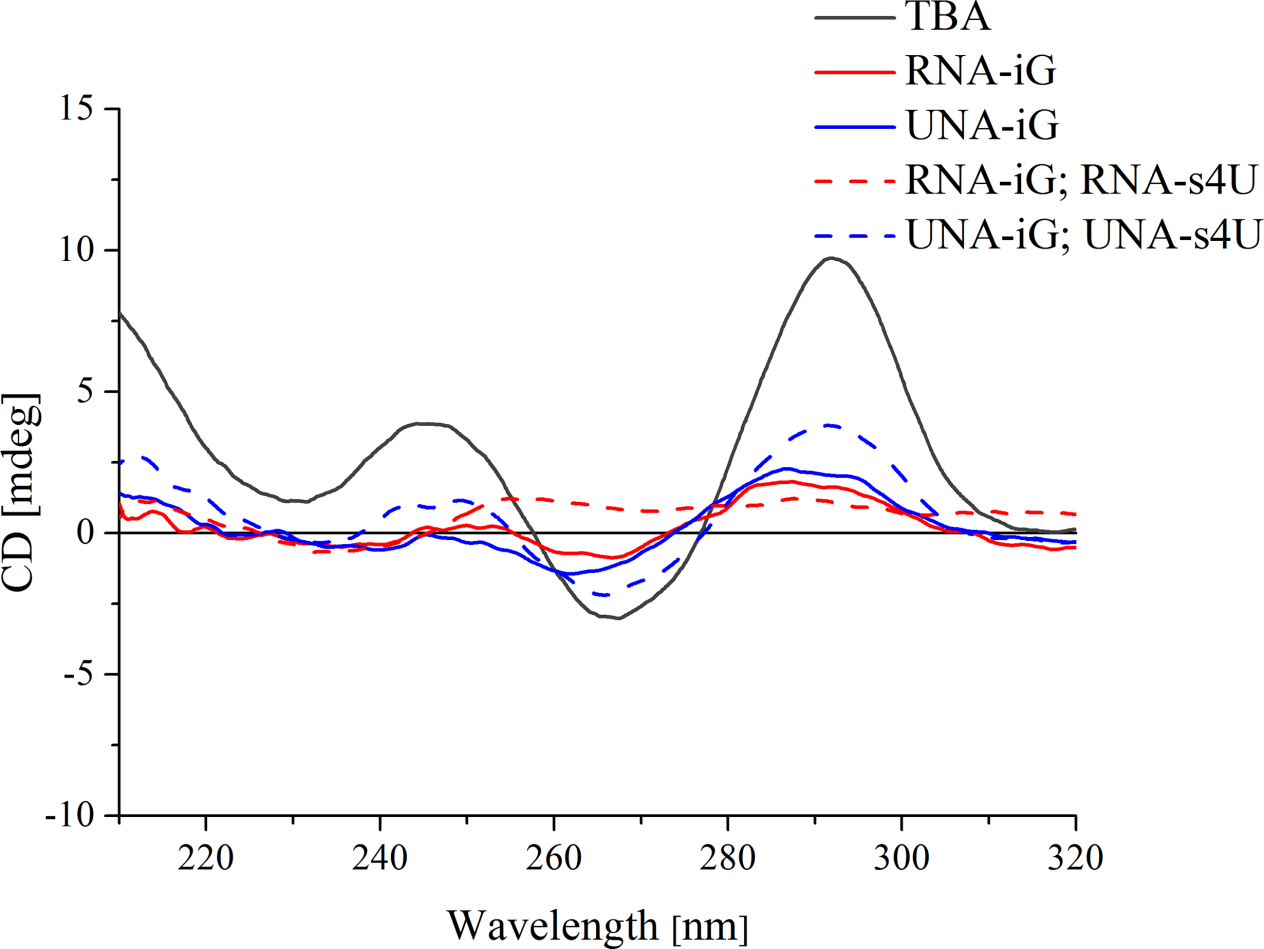
Representative circular dichroism spectra of TBA (black, solid line), and TBA variants substituted by RNA-iG (red, solid line), UNA-iG (blue, solid line), RNA-iG and RNA-s4U (red, dashed line), UNA-iG and UNA-s4U (blue, dashed line).

CD spectrum of unmodified TBA exhibits two positive bands *i*.*e*. a lower intensity peak near 245 nm and higher intensity band around 295 nm as well as negative signal near 265 nm (Fig 3). The CD pattern of TBA is consistent with previous reports indicating antiparallel G-quadruplex folding topology. On the contrary, the TBA variants substituted by UNA-iG or RNA-iG alone or in the presence of UNA-s4U or RNA-s4U exhibit a significant decrease in all CD band intensities or even loss of CD signals characteristic for antiparallel G-quadruplex. This shows that UNA-iG as well as RNA-iG has a negative influence on G-quadruplex formation, as indicated by thermodynamic analysis.

### Thermal difference spectra

**Fig 4:**
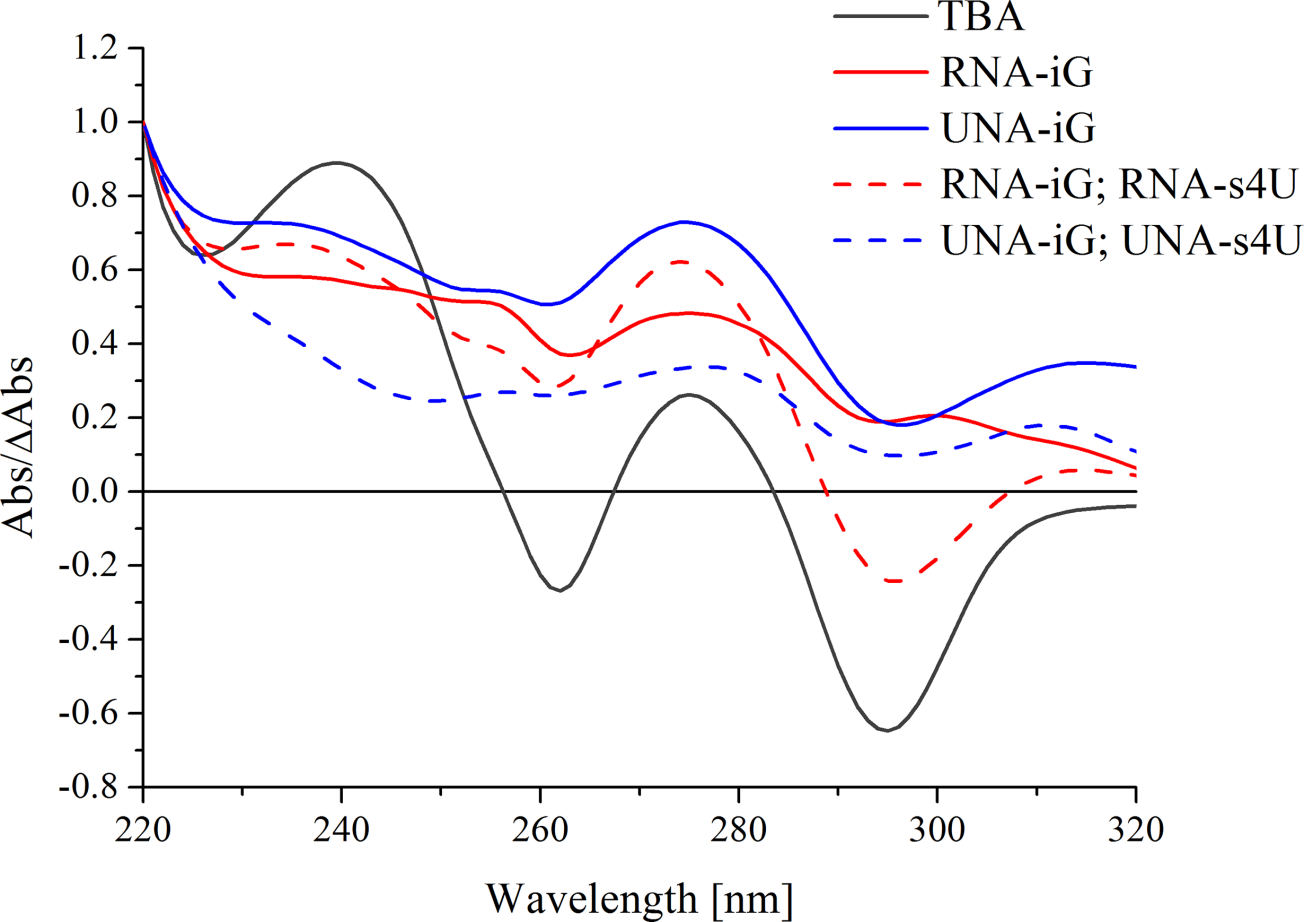
Representative thermal difference spectra of TBA (black, solid line), and TBA variants substituted by RNA-iG (red, solid line), UNA-iG (blue, solid line), RNA-iG and RNA-s4U (red, dashed line), UNA-iG and UNA-s4U (blue, dashed line).

Thermal difference spectra (TDS) are often used to supplement circular dichroism studies. This is a fast and cost-effective method to analyze differences in UV spectra. Absorbance values of nucleic acid structures below and above the melting point correspond to folded and unfolded nucleic acid state, respectively [23]. The subtraction of low temperature spectra from the high temperature spectra results in the pattern which is specific for a given nucleic acid structure. TDS of unmodified TBA exhibits a pattern characteristic for antiparallel G-quadruplex with two positive bands near 240 nm and 270 nm and two negative signals around 260 nm and 295 nm (Fig 4). Analogously to CD spectra, the TDS bands were significantly flattened for TBA variants containing only UNA-iG or RNA-iG substitution as well as for multiple substitutions of oligonucleotides with UNA-iG or RNA-iG with the addition of UNA-s4U or RNA-s4U, thereby confirming CD analysis and thermodynamic results.

### Thrombin time assay

**Fig 5:**
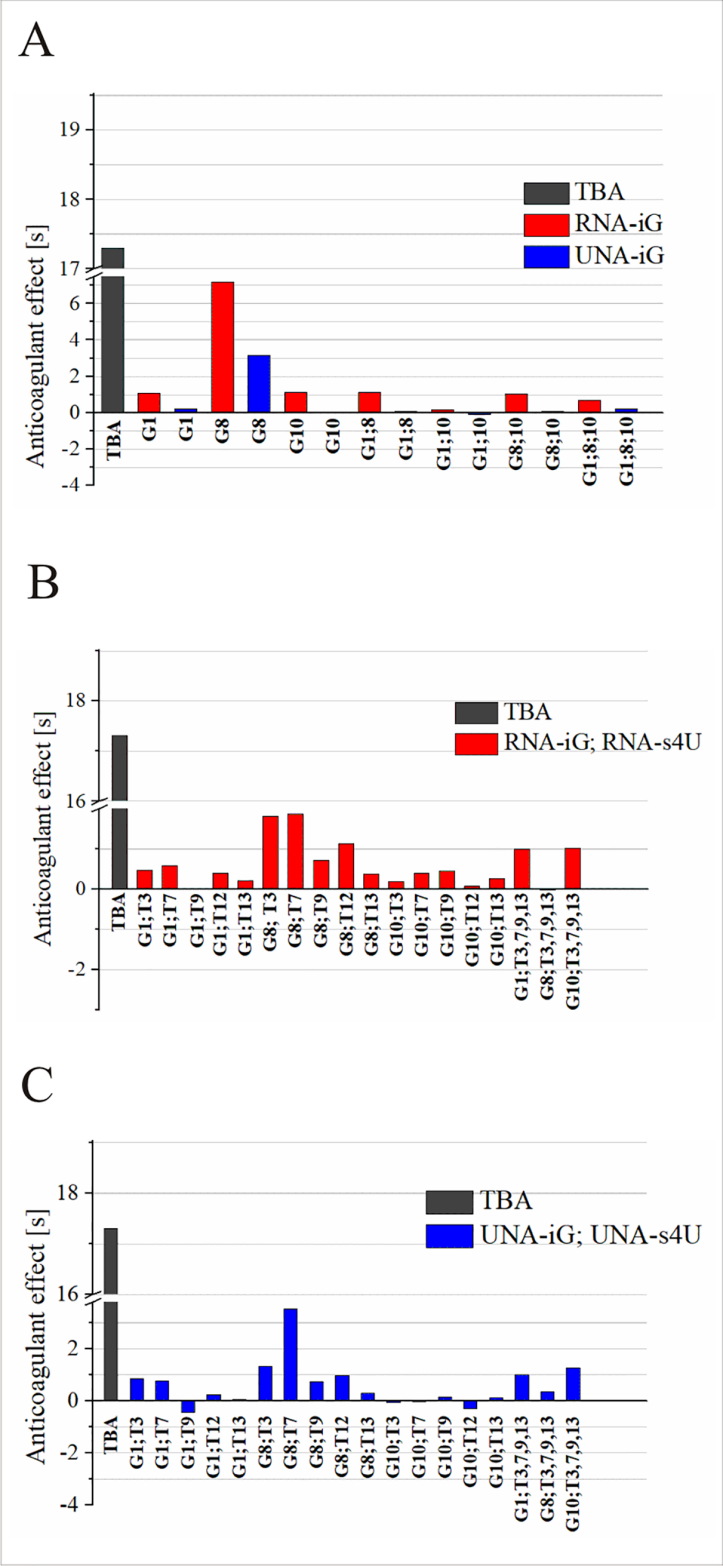
Anticoagulant activity of TBA variants modified with RNA-iG or UNA-iG (A), RNA-iG and RNA-s4U (B), UNA-iG and UNA-s4U (C).

Thrombin time assay is a commonly known test to diagnose blood coagulation disorders but it is also routinely used for analysis of anticoagulant potential of different compounds. Thrombin time (TT) is the time needed to form a clot within plasma samples after adding an excess of thrombin. The differences in thrombin time of plasma with and without anticoagulant can be named anticoagulation effect (AE). Despite the fact that previous report indicated that the presence of 2′-deoxy-isoguanosine improves TBA-thrombin interactions [10], our studies show that isoguanosine analogues in UNA and RNA series do not have such potential. All analyzed TBA variants containing UNA-iG or RNA-iG appeared to be poor anticoagulants exhibiting AE values that were at least 2.4 times lower compared to unmodified TBA (Fig 5 and S2 Table). The further addition of UNA-s4U or RNA-s4U did not result in the improvement of TBA anticoagulant properties. The results clearly indicate that the ability to form G-quartet is pivotal for the anticoagulant properties of the aptamer. Some published works also designate G-tetrad formation as the crucial factor for TBA activity [33,34]. It was previously reported that the analogues of guanine, such as 7-deazaguanine, evidently decrease thrombin binding as the residues are unable to form H-bonds within G-tetrad and consequently disrupt TBA structure [33,35]. On the other hand, there are examples of TBA variants, modified within loop region, that show considerable inhibitory activity despite the low thermodynamic stability [36]. In this case, however, it seems the thrombin acts as a molecular chaperone by stabilizing G-quadruplex structure and consequently an anticoagulant activity of TBA is retained [36,37]. Importantly, the formation of thermodynamically stable G-quadruplex structure of TBA with disrupted loop geometry could be detrimental for TBA anticoagulant properties [38]. Taken together, the literature data indicate that thermodynamic stability is not the only factor that determines inhibitory activity of TBA. Nevertheless, the results presented herein as well as above mentioned literature suggests that G-quartet formation is one of the major factors required for retaining TBA anticoagulant properties.

The studies described herein, clearly demonstrate that the presence of isoguanosine in both UNA and RNA series is detrimental for G-quadruplex formation and biological function of TBA. Substitution at positions G^1^, G^8^ or G^10^ significantly decreases thermodynamic stability of G-quadruplex structure. CD and TDS analyses indicate that modified monomer substitution is unfavorable for G-quadruplex folding. These substitutions promoted a significant decrease in the anticoagulant properties of TBA variants compared to unmodified TBA. This study enhances our understanding of how UNA-iG and RNA-iG affect the thermodynamics and biological function of TBA. This study also shows influence of novel isoguanine derivative of UNA on the thermodynamic stability and folding topology of G-quadruplexes in general.

## Supporting information

S1 File Chemical synthesis of UNA-isoguanine phosphoramidite. (DOCX)

S1 Table Thermodynamic parameters of intermolecular G-quadruplex formation of TBA variants modified with UNA-iG (iG^U^) and UNA-s4U (s4U^U^). (DOCX)

S2 Table The anticoagulant properties of TBA and modified TBA variants. (DOCX)

S3 Table Thermodynamic parameters of G-quadruplex formation of TBA variants modified with RNA-iG (iG^R^) or UNA-iG (iG^U^). (DOCX)

S4 Table Thermodynamic parameters of G-quadruplex formation of TBA variants modified with RNA-iG and RNA-s4U(iG^R^,s4U^R^) or UNA-iG and UNA-s4U (iG^U^,s4U^U^). (DOCX)
