# Supplementary material for: Novel isoguanine derivative of unlocked nucleic acid—Investigations of thermodynamics and biological potential of modified thrombin binding aptamer
Source: PLoS One. 2018 May 24;13(5):e0197835. doi: 10.1371/journal.pone.0197835 (PMC5967839; doi:10.1371/journal.pone.0197835)
Supplement: S1 File — (DOCX) [file pone.0197835.s001.docx]

**S1 File. Chemical synthesis of UNA-isoguanine phosphoramidite**

**Isoguanosine (2)**

2,6-Diaminopurine riboside (**1**; 2.80 g, 10 mmol) was dissolved in 98 mL of water, then LiNO_2_ (3.44 g, 65 mmol) was added and the mixture was heated up to 90°C until all reagents were dissolved. Afterwards, the solution was cooled to 70°C and 5.76 mL (80 mmol) of acetic acid was added dropwise. The mixture was cooled to rt and stirred for 5 minutes. The reaction mixture was evaporated to dryness, co-evaporated with methanol and toluene. The residue was suspended in methanol, filtered and dried at 55°C overnight to afford a powder of compound **2** (1.62 g, 57%). ^1^H NMR (DMSO-d_6_): δ 7.93 (s, 1H, H8), 5.64 (d, 1H, H1′), 5.37 (d, 1H, 2′OH), 5.11 (d, 1H, 3′OH), 4.52 (d, 1H, H2′), 4.07 (m, 1H, H3′), 3.92 (m, 1H, H4′), 3.62-3.49 (m, 2H, H5′); ^13^C NMR (DMSO-d_6_): δ 155.27 (C4), 138.48 (C8), 109.70 (C5), 87.61 (C1′), 86.97 (C4′), 72.86 (C2′), 70.76 (C3′), 61.79 (C5′).

**N^6^-[(Dimethylamino)methylene]isoguanosine (3)**

Nucleoside **2** (1.62 g, 5.7 mmol) was suspended in 30 mL of anhydrous DMF and 4.87 mL (28.5 mmol) of N,N-dimethylformamide dimethyl acetal was added. The mixture was stirred at rt overnight. The reaction mixture was evaporated to dryness to yield compound **3** (1.83 g, 95%) as an oil. ^1^H NMR (DMSO-d_6_): δ 9.18 (s, 1H, N=CH dmm), 8.08 (s, 1H, H8), 5.68 (d, 1H, H1′), 4.57 (t, 1H, H2′), 4.11 (m, 1H, H3′), 3.94 (m, 1H, H4′), 3.65-3.53 (m, 2H, H5′), 3.22 (s, 3H, CH_3_ dmm), 3.11 (s, 3H, CH_3_ dmm); ^13^C NMR (DMSO-d_6_): δ 161.37 (CH dmm), 157.19 (C4), 156.31 (C2), 154.67 (C6), 140.70 (C8), 113.61 (C5), 87.67 (C1′), 85.94 (C4′), 72.84 (C2′), 70.69 (C3′), 61.73 (C5′), 41.17 (CH_3_ dmm), 34.39 (CH_3_ dmm).

**N^6^-[(Dimethylamino)methylene]-5′-O-(4,4′-dimethoxytrityl)isoguanosine (4)**

Nucleoside **3** (1.93 g, 5.7 mmol) was dissolved in anhydrous pyridine (30 mL) and 4,4′-dimethoxytrityl chloride (1.93 g, 5.7 mmol) was added. Reaction mixture was stirred for 1.5 h at rt. Next, the solution was concentrated, dichloromethane was added and all was washed with saturated aqueous solution of sodium bicarbonate (50 mL). The organic phase was separated, dried over Na_2_SO_4_ and evaporated to dryness. The crude product was purified by silica gel column chromatography (0 - 10% methanol) to obtain compound **4** (3.03 g, 83%). ^1^H NMR (DMSO-d_6_): δ 11.07 (s, 1H, NH), 9.15 (s, 1H, N=CH dmm), 7.96 (s, 1H, H8), 7.36-6.82 (m, 13H, DMT), 5.74 (d, 1H, H1′), 5.55 (s, 1H, 2′OH), 5.15 (s, 1H, 3′OH), 4.49 (m, 1H, H2′), 4.20 (m, 1H, H3′), 4.00 (m, 1H, H4′), 3.72 (s, 6H, CH_3_O DMT), 3.19 (s, 3H, CH_3_ dmm), 3.17 (m, 2H, H5′), 3.09 (s, 3H, CH_3_ dmm); ^13^C NMR (DMSO-d_6_): δ 161.67 (CH dmm), 157.19 (C4), 154.90 (C2), 145.32 (C6), 140.16 (C8), 135.99, 136.06, 130.22, 130.13, 128.28, 128.19, 127.11 (Ar DMT), 113.61 (C5), 87.67 (C1′), 85.94 (C4′), 72.84 (C2′), 70.69 (C3′), 64.30 (C5′), 55.46, 55.45 (CH_3_O DMT), 41.57 (CH_3_ dmm), 34.80 (CH3 dmm).

**N^6^-[(Dimethylamino)methylene]-5′-O-(4,4′-dimethoxytrityl)-2′,3′-seco-isoguanosine (5)**

Nucleoside **4** (2.16 g, 3.3 mmol) was dissolved in a mixture of 1,4-dioxane (46 mL) and water (9.3 mL). Then NaIO_4_ (0.85 g, 3.97 mmol) in water (9.3 mL) was added under stirring at rt. During 1 h of the reaction the white precipitate was formed. Additional portion of 1,4-dioxane (43 mL) was added and after 15 min of stirring the suspension was filtered and the filter cake was washed with dioxane. The filtrates were combined, NaBH_4_ (139 mg, 3.69 mmol) was added, and the mixture was stirred for 30 min at rt. The reaction mixture was neutralized by the addition of pyridine/acetic acid mixture (1:1, v/v, ca. 7 mL). The mixture was concentrated, 50 mL of dichloromethane was added and washed with saturated aqueous solution of sodium bicarbonate (50 mL). The organic phase was separated, dried over Na_2_SO_4_ and evaporated to dryness to afford compound **5** (1.68 g, 79%) as a powder. ^1^H NMR (DMSO-d_6_): δ 11.06 (s, 1H, NH), 9.21 (s, 1H, N=CH dmm), 8.09 (s, 1H, H8), 7.25-6.77 (m, 13H, DMT), 5.70 (dt, 1H, H1′), 5.24 (s, 1H, 2′OH), 4.79 (s, 1H, 3′OH), 3.91-3.86 (m, 3H, H2′/4′), 3.70 (s, 6H, OCH_3_), 3.41 (d, 2H, H3′), 3.20 (s, 3H, CH_3_ dmm), 3.11 (s, 3H, CH_3_ dmm), 2.87-2.75 (m, 2H, H5′); ^13^C NMR (DMSO-d_6_): δ 161.25, 158.38, 157.85, 156.79, 154.14, 144.74 (Ar), 140.02 (C8), 135.80, 135.63 (Ar), 129.46, 127.67, 126.43, 113.03, 113.01(Ar-DMT), 84.99 (CH dmm), 82.96 (C1′), 79.67 (C4′), 63.8 (C5′), 61.58 (C2′), 60.80 (C3′), 54.96 (OCH_3_ DMT), 41.09 (CH_3_ dmm), 34.27 (CH_3_ dmm).

**N^6^-[(Dimethylamino)methylene]-2′-O-benzoyl-5′-O-(4,4′-dimethoxytrityl)-2′,3′-seco-isoguanosine (6)**

Nucleoside **5** (1.68 g, 2.6 mmol) was dissolved in anhydrous pyridine (2.1 mL) along with anhydrous dichloromethane (70 mL). The reaction mixture was stirred, cooled to -70°C and benzoyl chloride (331.9 µl, 2.86 mmol) was added portionwise over 20 min. The reaction was continued at -70°C for 3 h, the resulting solution was warmed to rt and ethanol (3.4 mL) was added. The solution was concentrated, dichloromethane (50 mL) was added and washed with saturated aqueous solution of sodium bicarbonate (50 mL). The organic phase was separated, dried over Na_2_SO_4_ and evaporated to dryness. The crude product was purified by silica gel column chromatography (0-2% methanol in dichloromethane) to yield nucleoside **6** (1.85 g, 75%). ^1^H NMR (DMSO-d_6_): δ 8.90 (s, 1H, N=CH dmm), 8.51 (s, 1H, H8), 8.14-6.79 (m, 18H, Ar), 6.35-6.27 (dt, 1H, H1′), 4.98-4.82 (m, 3H, H2′, H3′, 3′OH), 3.86 (m, 1H, H4′), 3.72 (s, 6H, OCH_3_), 3.48 (m, 2H, H3′), 3.20 (s, 3H, CH_3_ dmm), 3.16 (s, 3H, CH_3_ dmm), 2.97-2.83 (m, 2H, H5′); ^13^C NMR (DMSO-d_6_): δ 166.50, 165.00, 164.11, 158.58, 157.89, 156.04, 155.45, 150.88, 144.65, 141.69 (Ar), 139.8 (C8), 134.60, 134.40, 134.30, 133.60, 129.60, 129.10, 128.70, 127.70, 127.60, 113.02(Ar), 81.47 (C1′), 80.00 (C4′), 63.90 (C5′), 63.50 (C2′), 61.01 (C3′), 55.00 (OCH_3_ DMT), 41.00 (CH_3_ dmm), 34.80 (CH_3_ dmm).

**N^6^-[(Dimethylamino)methylene]-2′-O-benzoyl-3′-O-(2-cyanoethoxy(diisopropylamino)-phosphino)-5′-O-(4,4′-dimethoxytrityl)-dimethylformamide-2′,3′-seco-isoguanosine (7)**

Nucleoside **6** (506 mg, 0.69 mmol) along with tetrazole (48 mg, 0.69 mmol) was dissolved in 10 mL of anhydrous acetonitrile and 2-cyanoethyl-*N*,*N*,*N*′,*N*′-tetraisopropylphosphordiamidite (250 mg, 0.83 mmol) was added. The reaction mixture was stirred overnight at rt. The reaction mixture was diluted with 20 mL of dichloromethane containing 1% triethylamine and washed with saturated aqueous solution of sodium bicarbonate (20 mL). The organic phase was separated, dried over Na_2_SO_4_ and evaporated to dryness. The crude product was purified by silica gel column chromatography (0 - 80% ethyl acetate/hexane with addition of 1% of triethylamine) to yield amidite **7** (405 mg, 62%).

^31^P NMR (CD_3_CN): δ 145.86, 145.80.
