# Supplementary material for: Novel isoguanine derivative of unlocked nucleic acid—Investigations of thermodynamics and biological potential of modified thrombin binding aptamer
Source: PLoS One. 2018 May 24;13(5):e0197835. doi: 10.1371/journal.pone.0197835 (PMC5967839; doi:10.1371/journal.pone.0197835)
Supplement: S1 Table — (DOCX) [file pone.0197835.s002.docx]

**S1 Table.** Thermodynamic parameters of intermolecular G-quadruplex formation of TBA variants modified with UNA-iG (**iG^U^**) and UNA-s4U (**s4U^U^**).^a^

| **Position of**  **modification** | **Sequence**  **(5ʹ-3ʹ)** | **Average of curve fits** | | | |  | **T_M_^-1^ versus log C_T_ plots**  M versus log CT plots | | | | |
| --- | --- | --- | --- | --- | --- | --- | --- | --- | --- | --- | --- |
|  |  | **-ΔH˚ (kcal/mol)** | **-ΔS˚**  **(eu)** | **ΔG˚_37_ (kcal/mol)** | **T_M_**  **(˚C)** |  | **-ΔH˚**  **(kcal/mol)** | **-ΔS˚**  **(eu)** | **ΔG˚_37_ (kcal/mol)** | **T_M_**  **(˚C)** | **ΔT_M_**  **(˚C)** |
|  | GGTTGGTGTGGTTGG | 41.2±0.9 | 127.2±2.7 | -1.74±0.02 | 50.7 |  |  |  |  |  | 0 |
| T^7^, G^10^ | GGTTGG**s4U^U^**GT**iG^U^**GTTGG | 51.0±2.9 | 151.9±10.1 | -3.87±0.25 | 26.4 |  | 57.5±1.6 | 174.2±5.3 | -3.45±0.10 | 25.4 | -24.3 |
| T^9^, G^10^ | GGTTGGTG**s4U^U^iG^U^**GTTGG | 51.3±4.0 | 155.3±13.5 | -3.09±0.16 | 22.1 |  | 46.2±2.9 | 138.3±9.7 | -3.32±0.16 | 21.9 | -28.6 |
| G^10^, T^13^ | GGTTGGTiGT**iG^U^**GT**s4U^U^**GG | 51.2±2.0 | 155.4±6.7 | -2.95±0.17 | 21.3 |  | 56.2±8.5 | 172.7±29.1 | -2.63±0.54 | 21.1 | -29.4 |
| G^1^, T^3^, T^7^, T^9^, T^13^ | **iG^U^**G**s4U^U^**TGG**s4U^U^**G**s4U^U^**GGT**s4U^U^**GG | 48.6±8.9 | 143.6±30.0 | -4.01±0.49 | 26.7 |  | 47.6±4.1 | 140.7±13.7 | -3.95±0.21 | 26.1 | -24.0 |
| T^3^, T^7^, G^8^, T^9^, T^13^ | GG**s4U^U^**TGG**s4U^U^iG^U^s4U^U^**GGT**s4U^U^**GG | 52.6±7.1 | 155.3±23.5 | -4.39±0.17 | 29.6 |  | 45.9±0.7 | 133.3±2.4 | -4.52±0.03 | 29.4 | -21.1 |
| T^3^, T^7^, T^9^, G^10^, T^13^ | GG**s4U^U^**TGG**s4U^U^**G**s4U^U^iG^U^**GT**s4U^U^**GG | 58.3±1.7 | 175.2±5.7 | -3.98±0.15 | 28.2 |  | 51.9±4.9 | 153.9±16.3 | -4.19±0.19 | 28.4 | -22.5 |

^a^ buffer: 100mM KCl, 20mM sodium cacodylate, 0.5 mM EDTA(Na)2, pH 7.0
