# Supplementary material for: Novel isoguanine derivative of unlocked nucleic acid—Investigations of thermodynamics and biological potential of modified thrombin binding aptamer
Source: PLoS One. 2018 May 24;13(5):e0197835. doi: 10.1371/journal.pone.0197835 (PMC5967839; doi:10.1371/journal.pone.0197835)
Supplement: S2 Table — (DOCX) [file pone.0197835.s003.docx]

**S2 Table.** The anticoagulant properties of TBA and

modified TBA variants.

| **Position of modification** | **Sequence**  **(5ʹ-3ʹ)** | **EA**  **[s]** |
| --- | --- | --- |
|  | GGTTGGTGTGGTTGG | 17.30 |
| G^1^ | **iG^R^**GTTGGTGTGGTTGG | 1.07 |
| G^1^ | **iG^U^**GTTGGTGTGGTTGG | 0.21 |
| G^8^ | GGTTGGT**iG^R^**TGGTTGG | 7.17 |
| G^8^ | GGTTGGT**iG^U^**TGGTTGG | 3.15 |
| G^10^ | GGTTGGTGT**iG^R^**GTTGG | 1.10 |
| G^10^ | GGTTGGTGT**iG^U^**GTTGG | 0.04 |
| G^1^, G^8^ | **iG^R^**GTTGGT**iG^R^**TGGTTGG | 1.13 |
| G^1^, G^8^ | **iG^U^**GTTGGT**iG^U^**TGGTTGG | 0.08 |
| G^8^, G^10^ | GGTTGGT**iG^R^**T**iG^R^**GTTGG | 1.04 |
| G^8^, G^10^ | GGTTGGT**iG^U^**T**iG^U^**GTTGG | 0.08 |
| G^1^, G^10^ | **iG^R^**GTTGGTGT**iG^R^**GTTGG | 0.17 |
| G^1^, G^10^ | **iG^U^**GTTGGTGT**iG^U^**GTTGG | -0.12 |
| G^1^, G^8^, G^10^ | **iG^R^**GTTGGT**iG^R^**T**iG^R^**GTTGG | 0.70 |
| G^1^, G^8^, G^10^ | **iG^U^**GTTGGT**iG^U^**T**iG^U^**GTTGG | 0.20 |
| G^1^, T^3^ | **iG^R^**G**s4U^R^**TGGTGTGGTTGG | 0.46 |
| G^1^, T^3^ | **iG^U^**G**s4U^U^**TGGTGTGGTTGG | 0.85 |
| G^1^, T^7^ | **iG^R^**GTTGG**s4U^R^**GTGGTTGG | 0.57 |
| G^1^, T^7^ | **iG^U^**GTTGG**s4U^U^**GTGGTTGG | 0.75 |
| G^1^, T^9^ | **iG^R^**GTTGGTG**s4U^R^**GGTTGG | -0.01 |
| G^1^, T^9^ | **iG^U^**GTTGGTG**s4U^U^**GGTTGG | -0.45 |
| G^1^, T^12^ | **iG^R^**GTTGGTGTGG**s4U^R^**TGG | 0.40 |
| G^1^, T^12^ | **iG^U^**GTTGGTGTGG**s4U^U^**TGG | 0.22 |
| G^1^, T^13^ | **iG^R^**GTTGGTGTGGT**s4U^R^**GG | 0.20 |
| G^1^, T^13^ | **iG^U^**GTTGGTGTGGT**s4U^U^**GG | 0.07 |
| T^3^, G^8^ | GG**s4U^R^**TGGT**iG^R^**TGGTTGG | 1.80 |
| T^3^, G^8^ | GG**s4U^U^**TGGT**iG^U^**TGGTTGG | 1.32 |
| T^7^, G^8^ | GGTTGG**s4U^R^iG^R^**TGGTTGG | 1.87 |
| T^7^, G^8^ | GGTTGG**s4U^U^iG^U^**TGGTTGG | 3.53 |
| G^8^, T^9^ | GGTTGGT**iG^R^s4U^R^**GGTTGG | 0.71 |
| G^8^, T^9^ | GGTTGGT**iG^U^s4U^U^**GGTTGG | 0.74 |
| G^8^, T^12^ | GGTTGGT**iG^R^**TGG**s4U^R^**TGG | 1.13 |
| G^8^, T^12^ | GGTTGGT**iG^U^**TGG**s4U^U^**TGG | 0.97 |
| G^8^, T^13^ | GGTTGGT**iG^R^**TGGT**s4U^R^**GG | 0.37 |
| G^8^, T^13^ | GGTTGGT**iG^U^**TGGT**s4U^U^**GG | 0.30 |
| T^3^, G^10^ | GG**s4U^R^**TGGTGT**iG^R^**GTTGG | 0.19 |
| T^3^, G^10^ | GG**s4U^U^**TGGTGT**iG^U^**GTTGG | -0.05 |
| T^7^, G^10^ | GGTTGG**s4U^R^**GT**iG^R^**GTTGG | 0.40 |
| T^7^, G^10^ | GGTTGG**s4U^U^**GT**iG^U^**GTTGG | -0.03 |
| T^9^, G^10^ | GGTTGGTG**s4U^R^iG^R^**GTTGG | 0.45 |
| T^9^, G^10^ | GGTTGGTG**s4U^U^iG^U^**GTTGG | 0.15 |
| G^10^, T^12^ | GGTTGGTGT**iG^R^**G**s4U^R^**TGG | 0.07 |
| G^10^, T^12^ | GGTTGGTGT**iG^U^**G**s4U^U^**TGG | -0.30 |
| G^10^, T^13^ | GGTTGGTGT**iG^R^**GT**s4U^R^**GG | 0.25 |
| G^10^, T^13^ | GGTTGGTiGT**iG^U^**GT**s4U^U^**GG | 0.10 |
| G^1^, T^3^, T^7^, T^9^, T^13^ | **iG^R^**G**s4U^R^**TGG**s4U^R^**G**s4U^R^**GGT**s4U^R^**GG | 0.07 |
| G^1^, T^3^, T^7^, T^9^, T^13^ | **iG^U^**G**s4U^U^**TGG**s4U^U^**G**s4U^U^**GGT**s4U^U^**GG | 0.15 |
| T^3^, T^7^, G^8^, T^9^, T^13^ | GG**s4U^R^**TGG**s4U^R^iG^R^s4U^R^**GGT**s4U^R^**GG | -0.03 |
| T^3^, T^7^, G^8^, T^9^, T^13^ | GG**s4U^U^**TGG**s4U^U^iG^U^s4U^U^**GGT**s4U^U^**GG | 0.35 |
| T^3^, T^7^, T^9^, G^10^, T^13^ | GG**s4U^R^**TGG**s4U^R^**G**s4U^R^iG^R^**GT**s4U^R^**GG | 1.02 |
| T^3^, T^7^, T^9^, G^10^, T^13^ | GG**s4U^U^**TGG**s4U^U^**G**s4U^U^iG^U^**GT**s4U^U^**GG | 1.25 |
| **iG^U^** – UNA-iG, **iG^R^** – RNA-iG, **s4U^U^** – UNA-s4U **s4U^R^** – RNA-s4U | | |
