# Supplementary material for: Novel isoguanine derivative of unlocked nucleic acid—Investigations of thermodynamics and biological potential of modified thrombin binding aptamer
Source: PLoS One. 2018 May 24;13(5):e0197835. doi: 10.1371/journal.pone.0197835 (PMC5967839; doi:10.1371/journal.pone.0197835)
Supplement: S3 Table — (DOCX) [file pone.0197835.s004.docx]

**S3 Table.** Thermodynamic parameters of G-quadruplex formation of TBA variants

modified with RNA-iG (**iG^R^**) or UNA-iG (**iG^U^**).^a^

| **Position of**  **modification** | **Sequence**  **(5ʹ-3ʹ)** | **Average of curve fits** | | | |
| --- | --- | --- | --- | --- | --- |
|  |  | **-ΔH˚ (kcal/mol)** | **-ΔS˚**  **(eu)** | **ΔG˚_37_ (kcal/mol)** | **T_M_**  **(˚C)** |
|  | GGTTGGTGTGGTTGG | 41.2±0.9 | 127.2±2.7 | -1.74±0.02 | 50.7 |
| G^1^ | **iG^R^**GTTGGTGTGGTTGG | 25.6±3.8 | 87.3±13.6 | 1.49±0.48 | 19.9 |
| G^1^ | **iG^U^**GTTGGTGTGGTTGG | 27.9±3.7 | 95.0±12 | 1.55±0.07 | 20.7 |
| G^8^ | GGTTGGT**iG^R^**TGGTTGG | 35.3±1.0 | 112.3±3.2 | -0.51±0.02 | 41.6 |
| G^8^ | GGTTGGT**iG^U^**TGGTTGG | 35.4±1.0 | 114.8±3.2 | 0.21±0.01 | 35.1 |
| G^10^ | GGTTGGTGT**iG^R^**GTTGG | 23.7±5.5 | 77.5±17.9 | 0.39±0.05 | 31.9 |
| G^10^ | GGTTGGTGT**iG^U^**GTTGG | 37.1±9.3 | 125.0±30.5 | 1.65±0.33 | 23.8 |
| G^1^, G^8^ | **iG^R^**GTTGGT**iG^R^**TGGTTGG | 27.3±1.6 | 92.5±5.3 | 1.36±0.14 | 22.3 |
| G^1^, G^8^ | **iG^U^**GTTGGT**iG^U^**TGGTTGG | n.d. | n.d. | n.d. | <15.0 |
| G^8^, G^10^ | GGTTGGT**iG^R^**T**iG^R^**GTTGG | 32.4±2.3 | 105.4±7.4 | 0.31±0.01 | 34.1 |
| G^8^, G^10^ | GGTTGGT**iG^U^**T**iG^U^**GTTGG | n.d. | n.d. | n.d. | <15.0 |
| G^1^, G^10^ | **iG^R^**GTTGGTGT**iG^R^**GTTGG | n.d. | n.d. | n.d. | <15.0 |
| G^1^, G^10^ | **iG^U^**GTTGGTGT**iG^U^**GTTGG | n.d. | n.d. | n.d. | <15.0 |
| G^1^, G^8^, G^10^ | **iG^R^**GTTGGT**iG^R^**T**iG^R^**GTTGG | n.d. | n.d. | n.d. | <15.0 |
| G^1^, G^8^, G^10^ | **iG^U^**GTTGGT**iG^U^**T**iG^U^**GTTGG | n.d. | n.d. | n.d. | <15.0 |

^a^ buffer: 100 mM KCl, 20 mM sodium cacodylate, 0.5 mM EDTA(Na)2, pH 7.0;

n.d. – not determined
