# Supplementary material for: Novel isoguanine derivative of unlocked nucleic acid—Investigations of thermodynamics and biological potential of modified thrombin binding aptamer
Source: PLoS One. 2018 May 24;13(5):e0197835. doi: 10.1371/journal.pone.0197835 (PMC5967839; doi:10.1371/journal.pone.0197835)
Supplement: S4 Table — (DOCX) [file pone.0197835.s005.docx]

**S4 Table.** Thermodynamic parameters of G-quadruplex formation of TBA variants

modified with RNA-iG and RNA-s4U(**iG^R^,s4U^R^**) or UNA-iG and UNA-s4U (**iG^U^,s4U^U^**).^a^

| **Position of**  **modification** | **Sequence**  **(5ʹ-3ʹ)** | **Average of curve fits** | | | |
| --- | --- | --- | --- | --- | --- |
|  |  | **-ΔH˚ (kcal/mol)** | **-ΔS˚**  **(eu)** | **ΔG˚_37_ (kcal/mol)** | **T_M_**  **(˚C)** |
|  | GGTTGGTGTGGTTGG | 41.2±0.9 | 127.2±2.7 | -1.74±0.02 | 50.7 |
| G^1^, T^3^ | **iG^R^**G**s4U^R^**TGGTGTGGTTGG | 52.7±4.7 | 174.3±15.3 | 1.39±0.06 | 29.0 |
| G^1^, T^3^ | **iG^U^**G**s4U^U^**TGGTGTGGTTGG | 26.5±1.3 | 90.1±4.3 | 1.48±0.07 | 20.6 |
| G^1^, T^7^ | **iG^R^**GTTGG**s4U^R^**GTGGTTGG | 31.9±6.2 | 109.8±20.6 | 2.18±0.22 | 17.1 |
| G^1^, T^7^ | **iG^U^**GTTGG**s4U^U^**GTGGTTGG | 27.5±1.8 | 93.0±6.1 | 1.33±0.06 | 22.7 |
| G^1^, T^9^ | **iG^R^**GTTGGTG**s4U^R^**GGTTGG | 26.2±1.3 | 84.7±4.0 | 0.09±0.01 | 36.0 |
| G^1^, T^9^ | **iG^U^**GTTGGTG**s4U^U^**GGTTGG | 38.1±3.9 | 129.9±13.1 | 2.21±0.19 | 20.0 |
| G^1^, T^12^ | **iG^R^**GTTGGTGTGG**s4U^R^**TGG | 31.9±3.7 | 103.2±11.9 | 0.11±0.02 | 35.9 |
| G^1^, T^12^ | **iG^U^**GTTGGTGTGG**s4U^U^**TGG | 33.9±3.9 | 115.7±12.6 | 2.04±0.11 | 19.4 |
| G^1^, T^13^ | **iG^R^**GTTGGTGTGGT**s4U^R^**GG | n.d. | n.d. | n.d. | <15.0 |
| G^1^, T^13^ | **iG^U^**GTTGGTGTGGT**s4U^U^**GG | n.d. | n.d. | n.d. | <15.0 |
|  |  |  |  |  |  |
| T^3^, G^8^ | GG**s4U^R^**TGGT**iG^R^**TGGTTGG | 36.4±2.1 | 114.7±6.6 | -0.81±0.03 | 44.1 |
| T^3^, G^8^ | GG**s4U^U^**TGGT**iG^U^**TGGTTGG | 34.1±0.5 | 110.5±1.7 | 0.19±0.02 | 35.2 |
| T^7^, G^8^ | GGTTGG**s4U^R^iG^R^**TGGTTGG | 37.8±0.7 | 119.1±2.0 | -0.88±0.04 | 44.4 |
| T^7^, G^8^ | GGTTGG**s4U^U^iG^U^**TGGTTGG | 37.5±4.2 | 121.3±13.6 | 0.1±0.07 | 36.1 |
| G^8^, T^9^ | GGTTGGT**iG^R^s4U^R^**GGTTGG | 34.1±1.6 | 107.3±5.1 | -0.81±0.03 | 44.5 |
| G^8^, T^9^ | GGTTGGT**iG^U^s4U^U^**GGTTGG | 32.7±1.0 | 107.8±3.3 | 0.71±0.03 | 30.4 |
| G^8^, T^12^ | GGTTGGT**iG^R^**TGG**s4U^R^**TGG | 38.3±1.5 | 120.7±4.9 | -0.85±0.04 | 44.1 |
| G^8^, T^12^ | GGTTGGT**iG^U^**TGG**s4U^U^**TGG | 33.0±1.0 | 107.0±3.2 | 0.17±0.02 | 35.4 |
| G^8^, T^13^ | GGTTGGT**iG^R^**TGGT**s4U^R^**GG | 32.1±2.0 | 104.5±6.1 | 0.36±0.10 | 33.6 |
| G^8^, T^13^ | GGTTGGT**iG^U^**TGGT**s4U^U^**GG | 31.6±0.7 | 105.1±2.2 | 0.95±0.01 | 28.0 |
|  |  |  |  |  |  |
| T^3^, G^10^ | GG**s4U^R^**TGGTGT**iG^R^**GTTGG | 38.8±7.7 | 124.6±24.6 | -0.19±0.04 | 38.5 |
| T^3^, G^10^ | GG**s4U^U^**TGGTGT**iG^U^**GTTGG | 37.7±1.8 | 127.4±5.8 | 1.87±0.17 | 22.3 |
| T^7^, G^10^ | GGTTGG**s4U^R^**GT**iG^R^**GTTGG | 36.4±1.6 | 116.7±5.1 | -0.21±0.02 | 38.8 |
| T^9^, G^10^ | GGTTGGTG**s4U^R^iG^R^**GTTGG | 30.4±1.1 | 96.2±3.5 | -0.54±0.02 | 42.6 |
| G^10^, T^12^ | GGTTGGTGT**iG^R^**G**s4U^R^**TGG | 35.5±0.9 | 113.8±2.7 | -0.2±0.02 | 38.7 |
| G^10^, T^12^ | GGTTGGTGT**iG^U^**G**s4U^U^**TGG | 30.1±0.8 | 102.2±2.3 | 1.61±0.09 | 21.3 |
| G^10^, T^13^ | GGTTGGTGT**iG^R^**GT**s4U^R^**GG | 28.6±0.6 | 94.1±1.9 | 0.60±0.02 | 30.6 |
|  |  |  |  |  |  |
| G^1^, T^3^, T^7^, T^9^, T^13^ | **iG^R^**G**s4U^R^**TGG**s4U^R^**G**s4U^R^**GGT**s4U^R^**GG | 24.0±2.0 | 80.5±6.6 | 0.99±0.08 | 24.7 |
| T^3^, T^7^, G^8^, T^9^, T^13^ | GG**s4U^R^**TGG**s4U^R^iG^R^s4U^R^**GGT**s4U^R^**GG | 31.1±6.9 | 98.5±22.0 | -0.50±0.09 | 42.0 |
| T^3^, T^7^, T^9^, G^10^, T^13^ | GG**s4U^R^**TGG**s4U^R^**G**s4U^R^iG^R^**GT**s4U^R^**GG | 30.7±3.7 | 98.4±11.9 | -0.17±0.04 | 38.7 |
| G^1^, T^3^, T^7^, G^8^, T^9^, G^10^, T^13^ | **iG^R^**G**s4U^R^**TGG**s4U^R^iG^R^s4U^R^iG^R^**GT**s4U^R^**GG | n.d. | n.d. | n.d. | <15 |
| G^1^, T^3^, T^7^, G^8^, T^9^, G^10^, T^13^ | **iG^U^**G**s4U^U^**TGG**s4U^U^iG^U^s4U^U^iG^U^**GT**s4U^U^**GG | n.d. | n.d. | n.d. | <15 |

^a^ buffer: 100mM KCl, 20mM sodium cacodylate, 0.5 mM EDTA(Na)_2_, pH 7.0;

n.d. – not determined
